# Palmitic acid intake in children on gluten-free and generalized diets before and after palm oil reformulation: nutritional and metabolic implications

**DOI:** 10.3389/fnut.2026.1842117

**Published:** 2026-07-16

**Authors:** Ilaria Stradi, Lucia Diani, Maria Luisa Forchielli

**Affiliations:** 1 Nutrition Consultant, Sassuolo, Modena, Italy; 2 Dietetics and Clinical Nutrition Service, Morgagni-Pierantoni Hospital, Forlì, Italy; 3 Department of Medical and Surgical Sciences (DIMEC), University of Bologna, Bologna, Italy; 4 Health Science and Technologies - Interdepartmental Center for Industrial Research (CIRI-SDV), University of Bologna, Bologna, Italy

**Keywords:** celiac disease, food, health, Mediterranean, palmitic acid

## Abstract

**Background:**

The nutritional profile of many gluten-free products can make it challenging for children with celiac disease to maintain a balanced and healthy diet. Among the saturated fatty acids, palmitic acid (C16:0) is often a major component and has been implicated in the modulation of inflammation, insulin resistance, and lipid metabolism. In this study, we evaluated palmitic acid intake and overall dietary quality among school-aged children following gluten-free or unrestricted diets, before and after the widespread adoption of “no palm oil” labeling.

**Methods:**

Using 24-h dietary recalls and food frequency questionnaires, we estimated palmitic acid consumption, macronutrient intake, and key health indicators, including body mass index (BMI) z-scores and the Homeostasis Model Assessment of Insulin Resistance (HOMA-IR). Data collected before 2015 and after 2019 were compared to evaluate the dietary and metabolic changes over time.

**Results:**

Overall, 124 individuals were included: 62 on a gluten-free diet and 62 on a gluten-containing diet. Each group had 31 participants assessed before 2015 and after 2019. Following 2019, the mean palmitic acid intake was significantly lower (*p* < 0.001), particularly among celiac children. After 2019, the main dietary sources of palmitic acid shifted toward natural food, particularly dairy products, consistent with a qualitative improvement in fat intake. Variations in sugar consumption among dietary groups may act as a proxy for broader dietary differences related to palmitic acid intake. The gluten-containing diet group reported higher sugar and fiber intakes. Sugar intake correlated positively with BMI z-scores, whereas palmitic acid intake showed an initial association with HOMA-IR (*p* < 0.05).

**Conclusion:**

These changes suggest a progressive alignment with the Mediterranean dietary model, a widely recognized healthy eating pattern, in both children with celiac disease and healthy controls. This shift supports the role of food reformulation, labeling initiatives, and nutritional education in promoting long-term health benefits.

## 1 Introduction

The relationship between nutrition and health has attracted increasing attention, particularly in children, given the rising prevalence of obesity, metabolic disorders, and food hypersensitivity in this population. Among dietary factors, saturated fatty acids (SFAs) have been associated with several chronic conditions, including type 2 diabetes, cardiovascular disease, intestinal inflammation, and immune dysregulation, potentially through mechanisms involving insulin resistance, alterations in gut microbiota, and low-grade systemic inflammation (1–6). Consequently, many dietary guidelines recommend limiting SFA intake to reduce the risk of cardiometabolic diseases (7–9). However, the health effects of individual SFAs remain incompletely understood, highlighting the need for further research to inform future dietary recommendations.

Palm oil is a major source of saturated fatty acids (SFAs), particularly palmitic acid, which accounts for approximately 44% of its fatty acid content (10). Owing to its stability, low cost, and favorable technological properties, palm oil is widely used in processed foods and has become one of the most consumed vegetable oils worldwide (11). Concerns regarding its health effects largely stem from its high palmitic acid content. Experimental and observational studies suggest that palmitic acid may promote low-grade inflammation, alter gut microbiota composition, and impair insulin sensitivity, particularly in individuals with obesity (12–15). Furthermore, a higher intake of saturated fats, especially palmitic acid, has been associated with increased cardiovascular and all-cause mortality in large population studies (16). These findings have contributed to the growing interest in the metabolic effects of palmitic acid and its role in overall dietary patterns.

Evidence from human observational and interventional studies suggests that lower palmitic acid exposure is generally associated with improved insulin sensitivity and lower HOMA-IR values. For instance, palmitate-enriched serum triglycerides correlate positively with HOMA-IR, circulating palmitic acid levels are associated with poorer glycemic and insulinemic markers, and experimental diets rich in palmitate impair insulin sensitivity compared to oleate-rich diets (17–19). However, palmitate may act as both a causal contributor to insulin resistance and as a marker of overall metabolic health. Indeed, dietary palmitic acid intake is not always strongly associated with insulin resistance, as endogenous palmitate production via *de novo* lipogenesis (driven by excess carbohydrate intake or caloric surplus) may play a substantial role. Therefore, we hypothesized that a lower dietary proportion of palmitic acid, as part of a more balanced dietary pattern consistent with the Mediterranean diet, would be associated with a lower HOMA-IR and a more favorable metabolic profile (20–22).

Public health efforts across Europe, including ingredient labeling regulations introduced by the European Parliament in 2014, have prompted manufacturers to reduce or remove palm oil from processed foods (23). These changes provide an opportunity to evaluate the influence of policy-driven reformulation on population-level exposure to saturated fatty acids.

Children following gluten-free diets (GFDs), primarily for celiac disease or non-celiac gluten sensitivity, represent a relevant subgroup for investigation. Many gluten-free processed products contain added fats, including palm oil, to replicate the functional and sensory properties of gluten. As a result, these children may have a higher intake of saturated fatty acids, including palmitic acid, with potential implications for metabolic health.

Against this background, the present study aimed to assess dietary exposure to palmitic acid in school-aged children by comparing those consuming a gluten-containing diet with those on a GFD before and after the widespread reformulation of processed foods following palm oil labeling regulations. Using the Mediterranean diet as a reference for optimal fat quality and metabolic balance, we also examined whether the observed dietary patterns reflected a shift toward this dietary model. As secondary objectives, we investigated the associations between palmitic acid intake and dietary, anthropometric, and metabolic parameters. Overall, these findings may improve our understanding of the impact of food reformulation on dietary fat quality and nutrient exposure in pediatric populations, providing evidence relevant to pediatric nutrition and public health.

## 2 Patients and methods

### 2.1 Study design

This observational study used data from a dynamic clinic-based longitudinal cohort. The source cohort included 441 subjects (281 patients and 160 controls). Comparisons were made between two periods: before 2015 (<2015), when palm oil was widely used, and after 2019 (>2019), when it had largely been replaced by other oils. Children were recruited from nutritional counseling (e.g., for guidance on meal planning, balanced dietary habits, transient constipation or minor episodes of gastroesophageal reflux) and celiac follow-up visits (on a gluten-free diet for >2 years). Based on an a priori power analysis (α = 0.05, power = 80%), a minimum of 31 subjects per group was required for the study. After 1:1 age- and gender-matching between subjects treated before 2015 and after 2019, 31 matched pairs were available and included in the final analysis.

All children included in the study lived in the same regional area, minimizing geographic differences in food access and dietary patterns. All children with celiac disease were considered adherent to a GFD with negative anti-transglutaminase IgA antibodies by ELISA (<7 U/mL) or deamidated gliadin peptide IgG antibodies by ELISA (<7 U/mL) in cases of serum immunoglobulin A deficiency, negative anti-endomysial antibodies, and the Biagi score of 3–4 (24).

Data were obtained under a study protocol approved by the Ethics Committee of the University Hospital Sant’Orsola Malpighi (38/17/O/Oss). The study was conducted in accordance with the Declaration of Helsinki, anonymity was guaranteed, and informed consent was obtained from parents at the time of enrolment.

#### 2.1.1 Dietary intake data

Dietary intake data consisted of a 24-h dietary recall administered by trained dietitians supported by a 7-day food frequency questionnaire (FFQ) to improve accuracy and capture habitual intakes. A comprehensive list of food items, including brand-name products, was reviewed by the participants, to improve specificity. The food was categorized into standard groups: vegetables, fruits, grains, protein foods, dairy, oils, solid fats, seasonings, beverages (plain or carbonated water, sodas, and juices), and sweets. Portion sizes were estimated using common kitchen utensils (e.g., measuring spoons and food scales) or portion size visualization tools to improve quantification accuracy.

Dietary data were analyzed at the time of collection using the European Institute of Oncology (IEO) food composition database (25) and processed using the Winfood 3 PRO software (last accessed august 2025^1^). Nutrient computations included the daily average intake of calories, macronutrients, simple sugars, and fiber. Palmitic acid intake was estimated based on database values, accounting for both natural and added sources (e.g., palm oil). The collected dietary data were assessed using the Reference Intake Levels of Nutrients and Energy for the Italian Population (LARN) recommendations as a reference standard (26).

#### 2.1.2 Anthropometric and laboratory parameters

The anthropometric data included weight (kg), height (cm), and body mass index (kg/m^2^) (BMI). Measurements were performed using the same healthcare tools and dressing conditions. The scale was calibrated before measurements. Children and adolescents were measured in a standing position without shoes using a stadiometer. If blood tests were available, two indices were computed: (1). the Homeostasis Model Assessment of insulin resistance (HOMA-IR) (computed based on fasting glucose mg/dL × insulin mU/L divided by 405), and (2). the triglyceride-glucose index (TyG) calculated using the natural logarithm of the mathematical product of fasting triglycerides (mg/dL) and fasting glucose (mg/dL), divided by two. Both indices are biomarkers used to screen for insulin resistance (27–30).

### 2.2 Statistical analysis

Descriptive statistics for continuous variables are presented as means and standard deviations (SD) or medians and interquartile ranges (IQR), as appropriate. Statistical analyses were conducted using Stata software (version 18 StataCorp, College Station, TX, United States).

A normality test was performed prior to the statistical analysis. Several continuous variables were transformed to normalize the distributions, reduce the influence of outliers, and stabilize the variance. Specifically, palmitic acid intake, caloric intake, and HOMA-IR were log-transformed, whereas sugar and fiber intakes were square-root transformed. BMI was converted into z-scores.

After establishing homogeneity of variance, ANOVA was used to test whether palmitic acid intakes differed between diet and time in the groups across the two time periods. Subsequently, regressions were performed (1). to evaluate the association between dietary variables and palmitic acid intake across groups and (2) to examine the association between dietary exposure and anthropometric parameters (BMI z-score), and insulin resistance (HOMA-IR, TyG). To assess the potential influence of extreme observations, we repeated the analyses using winsorized versions of the log-transformed palmitic acid variable. Winsorization was implemented in Stata using the winsor2 package with cuts at the 1st/99th and 5th/95th percentiles. Analyses reflected between-sample comparisons in age- and gender-matched cross-sectional groups rather than within-subject longitudinal changes. Statistical significance was set at *p* < 0.05.

## 3 Results

**FIGURE 1:**
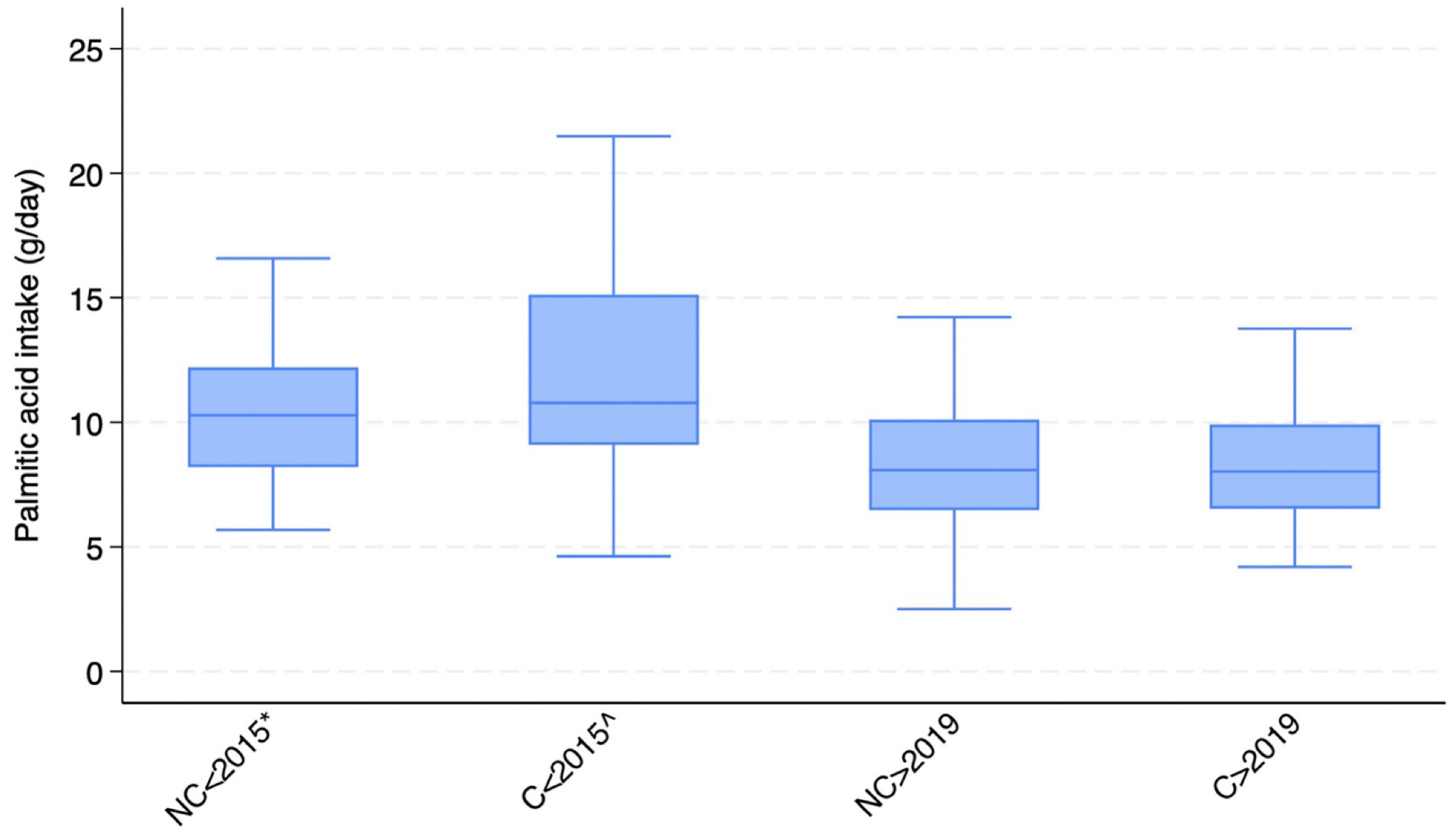
Summary of palmitic acid intake in the four groups. Palmitic acid intake was expressed in terms of medians and quartiles (25th–75th percentiles) in the four groups. <2015, time when palm oil was normally added to food; >2019, period when palm oil addition to food was reduced; NC, children consuming a gluten-containing diet. (C) Children with celiac disease on a gluten-free diet. *p < 0.05, subjects evaluated before 2015 (NC < 2015) had a greater amount of palmitic acid than the subjects evaluated after 2019 (NC > 2019). ^∧^*p* < 0.005, the celiac group (C < 2015) had a significantly higher amount of palmitic acid than the C > 2019 group.

A total of 124 individuals were included: 62 on a gluten-free diet and 62 on a gluten-containing diet, with each diet group comprising 31 subjects assessed before 2015 and 31 assessed after 2019. The descriptive data are presented in Table 1. Figure 1 shows the overall palmitic acid intakes in the four groups.

**TABLE 1:** Summary of the four groups.

Groups	Subjects (number)	Mean age ± standard deviation (years)	Female	Male
NC < 2015	31	10.2 ± 2.7	16	15
NC > 2019	31	10 ± 2.5	16	15
C < 2015	31	10 ± 2.6	15	16
C > 2019	31	10.5 ± 2	15	16

<2015, period when palm oil was commonly added to food; >2019, period when palm oil addition to food was reduced; NC, children consuming a gluten-containing diet; C, children with celiac disease on a gluten-free diet.

Fat intake and palmitic acid trends: The C < 2015 group had the highest palmitic acid intake (64% ± 10% of total saturated fat). After palm oil reduction in processed foods, the C > 2019 group showed a decreased palmitic acid intake, which aligned with that of the NC > 2019. Saturated fat intake in both groups met the LARN recommendations (10% of total energy) before 2015 and declined after 2019.

Nutrient intake and metabolic patterns: Only the NC > 2019 group met the LARN fiber recommendation (8.4 g/1,000 kcal), while the other groups remained below this threshold. The C groups showed low fiber intake throughout, significantly lower than NC post-2019 (*p* = 0.001). Simple sugar intake exceeded LARN’s 15% limit in the NC groups and in the C < 2015 group, while C > 2019 remained within limits. No differences were observed in the other macronutrients. The average HOMA-IR and TyG values did not differ across the groups. The Z-score BMI differed significantly between the NC groups (*p* < 0.05).

The dietary intake, anthropometric measurements, and metabolic values of the four groups are summarized in Tables 2, 3.

**TABLE 2:** Summary of dietary intakes, anthropometric measures, and metabolic values in the four groups.

Variables	Group NC < 2015			Group C < 2015			Group NC > 2019			Group C > 2019		
	Mean	SD	*N*	Mean	SD	*N*	Mean	SD	*N*	Mean	SD	*N*
Caloric intake (Kcal/d)	1,856	394	31	1,674	346	31	1,781	286.2	31	1,818	197	31
Carbohydrate intake (g/d)	242.7	68	31	210	68.8	31	236	53.8	31	234	62	31
Lipid intake (g/d)	66.5	21.4	31	63.3	21.7	31	63.5	16.9	31	70	17	31
Protein intake (g/d)	72	19.8	31	66.2	22.5	31	66.8	17	31	63	17	31
Palmitic acid (g/d)	10.2*	2.8	31	12.2^∧^	5	31	8.2	2.8	31	8.6	2.6	31
Saturated fatty acids (g/d)	23.1	8.1	31	19.3	8.6	31	18.8	7.8	31	19.6	5.4	31
Palmitic acid/saturated fatty acids (%)	46°	10	31	63.8^∧∧^	9.9	31	45	9.6	31	44.3	8.9	31
Fibers (g/d)	13.4	4.4	31	13.5	5	31	15.9#	4.3	31	12	4.7	31
Fibers/calories (gr per 1,000 Kcal)	7.4	2.9	31	8	2.6	31	9.1##	2.7	31	6.6	2.4	31
Simple sugars (g/d)	82.2	30.9	31	67	23	31	74.8###	27.6	31	52.3	19.8	31
Simple sugars/calories (%)	18	7.1	31	16^∧∧∧^	5.6	31	16.8####	5.5	31	11.5	4.2	31
HOMA-IR	1.6	0.8	21	1.6	0.95	28	1.7	0.9	16	1.5	1.4	29
Zscore BMI	−0.19**	1.5	31	−0.3	1.2	31	0.8	1.5	31	0.1	1.1	31

<2015, period when palm oil was commonly added to food; >2019, period when palm oil addition to food was reduced; NC, children consuming a gluten-containing diet; C, children with celiac disease on a gluten-free diet; SD, standard deviation; *N*, number of participants; Kcal, calories; HOMA-IR, Homeostasis Model Assessment of Insulin Resistance (glucose mg/dL × insulin mU/L /405); BMI, body mass index. Significant pairwise comparisons of means in the groups:

1. NC < 2015 vs. NC > 2019: *p < 0.05;

**p < 0.005.

2. C < 2015 vs. C > 2019: ^∧^*p* < 0.005; ^∧∧^*p* < 0.001; ^∧∧∧^*p* < 0.01.

3. NC < 2015 vs. C < 2015: °*p* < 0.001.

4. NC > 2019 vs. C > 2019: #*p* = 0.005; ##*p* < 0.001; ###*p* < 0.005; ####*p* < 0.005.

**TABLE 3:** Summary of triglyceride-glucose index and lipid profile in the four groups.

Variables	Group NC < 2015			Group C < 2015			Group NC > 2019			Group C > 2019		
	Mean	SD	*N*	Mean	SD	*N*	Mean	SD	*N*	Mean	SD	*N*
TyG index	7.6	0.4	23	7.7	0.3	31	7.6	0.3	21	7.5	0.4	31
Total cholesterol (mg/dL)	146.7	16	23	144	29	31	156	16	21	156	24	31
Triglycerides (mg/dL)	57	20	23	62	25	31	56	18	21	63	24	31
High density lipoprotein (mg/dL)	59	11	23	58	15	31	61	9	21	57.4	12	31

Tyg, triglyceride-glucose index.

### 3.1 Effects of diet and time on palmitic acid intake

A two-way ANOVA was conducted to assess the effects of diet type and time on log-transformed palmitic acid intake. The initial model with main-effects was significant, *F*(2, 121) = 11.48, *p* < 0.001, *R*^2^ = 0.160. A significant main effect of time was observed (*F* = 20.21, *p* < 0.001), whereas diet type was not a significant predictor (*F* = 2.74, *p* = 0.100). The interaction between diet and time was non-significant (*F* = 0.30, *p* = 0.583), suggesting that the temporal effect on palmitic acid intake was consistent across diet groups. Bonferroni-adjusted *post hoc* comparisons (based on the groups variable) showed a significant difference between NC > 2019 and NC < 2015 (*p* < 0.05), and between C > 2019 and C < 2015 (*p* < 0.005).

To account for the potential influence of outliers, the analysis was repeated using winsorized log-transformed values of palmitic acid. The revised model confirmed that time was a significant predictor (*p* < 0.001). Across all specifications, the results remained qualitatively similar to those obtained using the original log-transformed variables. In particular, the direction and magnitude of the estimated coefficients were stable. These findings suggest that the observed associations are robust and not driven by a small number of outliers. Diet type was significantly associated with palmitic acid levels after winsorization; however, this association was no longer present when sugar intake was excluded from the model. As sugar intake differed across dietary groups and was also associated with palmitic acid levels, it was considered a potential confounding factor. The loss of statistical significance after adjusting for sugar intake suggests that differences in sugar consumption may partly explain the observed differences in palmitic acid levels between the dietary groups. This pattern may reflect broader differences in dietary habits, whereby children on GFD consume a dietary pattern characterized by relatively higher intakes of total fat and saturated fatty acids, including palmitic acid, whereas those consuming a gluten-containing diet may have a greater intake of sugar-rich foods.

No other nutritional or metabolic covariates were significantly associated with palmitic acid levels in the adjusted model. Model diagnostics indicated low multicollinearity, with variance inflation factors (VIF ≈ 1.4) well below the conventional thresholds.

### 3.2 Secondary endpoints regression analyses: BMI and metabolic variables

To further explore the potential metabolic implications of the dietary regimens and associated changes in palmitic acid intake, secondary regression analyses were conducted using BMI and HOMA-IR or TyG as outcome variables. These analyses aimed to assess whether the differences in diet type and palmitic acid levels were related to body composition and insulin resistance.

#### 3.2.1 BMI z-score models

Linear regression was used to examine the effects of nutritional factors, diet type, and time on the BMI z-score. Time was a significant predictor (*p* < 0.05), with the NC > 2019 group showing higher BMI z-scores than the NC < 2015 (*p* < 0.05) and C < 2015 (*p* = 0.01) groups. Diet type and its interaction with time were not significant predictors. Among the dietary variables, sugar intake was positively associated with the BMI z-score (*p* < 0.05). Conversely, a higher proportion of palmitic acid within saturated fat was negatively associated with BMI z-score (*p* = 0.01), a counterintuitive finding that requires further exploration.

#### 3.2.2 HOMA-IR and TyG models

Linear regression models with log-transformed HOMA-IR as the outcome revealed an initial positive association between log-palmitic acid intake and log-HOMA-IR (*p* < 0.05). However, when diet type and sugar intake were introduced into the model as confounding factors, the independent associations of both palmitic acid and diet type with HOMA-IR were no longer statistically significant. This suggests that the observed effects of diet and palmitic acid are largely confounded or accounted for by sugar consumption, which is the primary driver of the association with HOMA-IR. This also emerged in the previous analysis. A weak, non-significant trend was observed between the BMI z-score and HOMA-IR. In contrast to the findings for HOMA-IR, when the analyses were repeated using the TyG index as the dependent variable, no statistically significant associations were observed with palmitic acid intake, diet type, or sugar consumption. This lack of association remained consistent across all model specifications, including those adjusted for potential confounding factors. Winsorization of key variables produced minimal changes in regression coefficients or statistical significance, supporting the robustness of the findings.

#### 3.2.3 *Post hoc* exploration: sugar × fiber interaction over HOMA-IR

**FIGURE 2:**
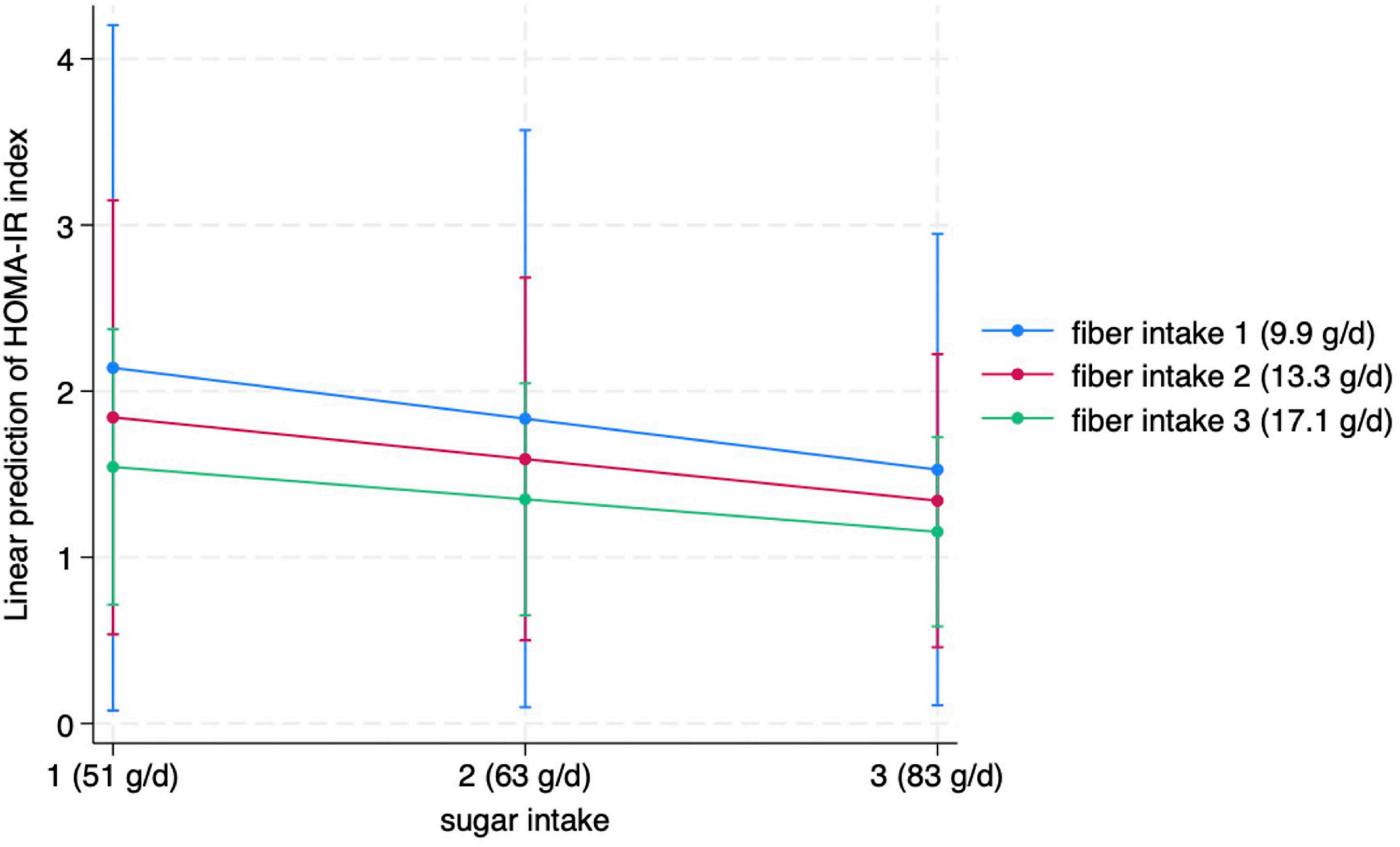
Prediction of HOMA-IR index at low, medium, and high levels of sugars and fiber intake. Fiber 1, 2, and 3 indicate the 25, 50th, and 75th percentiles of fiber intake. Sugar 1, 2, and 3 indicate the 25th, 50th, and 75th percentiles of sugar intake. Predictive margins with 95% CIs.

The multivariate analysis assessing the independent role of carbohydrates revealed a significant inverse association between sugar intake and HOMA-IR (*p* < 0.005). To further clarify this relationship, a *post hoc* margin analysis was conducted to evaluate the interaction between sugar and fiber intakes. The predicted values of HOMA-IR were estimated across low, medium, and high levels of both nutrients. Although the formal interaction term between sugar and fiber did not reach statistical significance, visual inspection of the marginal plots (Figure 2) showed that higher fiber intake progressively attenuated the increase in predicted HOMA-IR associated with increased sugar consumption.

## 4 Discussion

### 4.1 Dietary reformulation and palmitic acid intake

Over the past decade, public health initiatives and industry reformulations have contributed to changes in processed food composition, including reduced use of palm oil, a major dietary source of palmitic acid in children’s diets. To our knowledge, this study is among the first to evaluate temporal changes in dietary palmitic acid intake in children on a gluten-containing diet or GFD.

In this matched repeated cross-sectional study, we observed a significant reduction in palmitic acid intake after 2019, with a more pronounced decrease in the GFD group. Before 2015, palmitic acid accounted for over 60% of saturated fat intake in children on a GFD. No differences between groups were observed after 2019. These findings are consistent with reduced use of palm oil in processed foods and may reflect broader shifts in dietary fat sources at the population level. The observed reduction in industrially derived saturated fats is compatible with a shift toward diets richer in unsaturated fat sources such as olive oil, nuts, and dairy products. This pattern is consistent with dietary profiles such as the Mediterranean diet (20–22), although overall dietary adherence was not directly assessed.

Nut–cocoa spreads remained a relevant source of palmitic acid in both groups. Packaged snack consumption also remained higher among children on a GFD, possibly reflecting efforts to avoid gluten cross-contamination, particularly in school or out-of-home settings.

### 4.2 Sugar, fiber, and metabolic implications

Sugar intake was positively associated with BMI z-scores (*p* < 0.05), consistent with established associations between added sugar intake and adiposity. In contrast, a higher proportion of palmitic acid within total saturated fat was inversely associated with BMI z-scores (*p* = 0.01), which may reflect dietary substitution between fat-rich and sugar-rich processed foods rather than a direct protective effect.

Group comparisons showed that the post-2019 control group had higher BMI z-scores, sugar intake, and HOMA-IR compared with the corresponding GFD group. These differences may partly reflect lifestyle changes during the COVID-19 period, including reduced physical activity and altered eating behaviors. In contrast, the absence of similar changes in children with celiac disease may suggest that long-term dietary management and greater attention to food selection, nutritional labels and meal planning could contribute to more stable dietary behaviors, as shown in the literature (31, 32). Notably, many families of CD children reported increased home meal preparation during lockdown periods, which may have further supported healthier food choices in children. While this interpretation remains speculative, it raises the possibility that the dietary discipline associated with CD management may confer benefits beyond gluten avoidance. However, because lifestyle factors were not systematically assessed, these findings should be interpreted cautiously.

A notable finding was the differential behavior of insulin resistance indices. Dietary factors, including sugar and palmitic acid intake, were associated with log-HOMA-IR, whereas no consistent associations were observed with the TyG index. This may reflect differences in sensitivity to early metabolic alterations or limitations of these surrogate markers in pediatric populations.

The inverse association between sugar intake and HOMA-IR is unlikely to indicate a protective effect and may instead reflect reverse causation, residual confounding, reporting bias, or dietary modification following nutritional counseling. Exploratory analyses suggested that higher fiber intake may attenuate the association between sugar intake and metabolic risk, although this interaction did not reach statistical significance, likely due to limited power. These findings support a potential modulatory role of dietary fiber in pediatric glucose metabolism and warrant further investigation.

### 4.3 Gluten-free diets: from concern to opportunity

Historically, gluten-free diets (GFDs) have been considered nutritionally suboptimal, often characterized by lower fiber, iron, and B-vitamin contents and higher fat and sugar intakes (33–36). However, the analysis of the post-2019 matched sample suggested a more favorable dietary profile, characterized by improved fat quality and moderate sugar intake. Despite these improvements, the dietary fiber intake remains suboptimal. Fruit and vegetable consumption was also low, averaging approximately one portion per day for each food group, which may partly explain the observed inadequate fiber intakes.

Nevertheless, the increasing availability of gluten-free products made from pulses and nutrient-rich cereals or pseudocereals, such as buckwheat, quinoa, and teff, as well as the wider use of brown rice in pasta and baked goods, represents a nutritional improvement by increasing the dietary fiber content and reducing the glycemic impact. However, there remains room for improvement. Taken together, these findings highlight the importance of dietary counseling aimed at promoting overall dietary quality beyond gluten exclusion.

### 4.4 Methodological considerations and future directions

The combined use of 24-h dietary recalls and food frequency questionnaires strengthened the dietary assessment. However, the limitations include underreporting, inter-individual variability, and the absence of fully repeated within-subject measures.

Although the study was designed as a prospective observational cohort study, follow-up depended on attendance at routine clinical visits, resulting in a dynamic cohort. Consequently, the analyses primarily reflect repeated cross-sectional comparisons rather than true within-subject longitudinal changes within individuals. Therefore, causal inferences cannot be drawn from these observed temporal associations. Temporal differences may also have been influenced by secular dietary trends, changes in food availability, increased nutritional awareness, and other unmeasured confounders. However, the fact that the participants resided within the same regional area may have mitigated the impact of some of these factors.

Another limitation was the presence of missing data owing to changes in clinical practice over time. For example, insulin concentrations were not routinely collected during the earlier years of the study, whereas they are now commonly measured for the calculation of HOMA-IR and the tailoring of nutritional counseling. An additional limitation is the lack of physical activity data, which have not been routinely monitored in the past.

Limited information on the nutrient composition of gluten-free foods before 2019 may have also introduced measurement bias. Nevertheless, the consistency of the observed trends across the analyses supports the robustness of the findings.

Future research should investigate whether changes in dietary fat sources translate into measurable effects on gut microbiota composition, lipid metabolism, and inflammatory markers. Repeated dietary assessments are also required to better characterize individual dietary trajectories and their clinical implications. Such investigations would help clarify the mechanisms underlying the observed dietary changes and their potential effects on long-term health outcomes.

## 5 Conclusion

This study highlights a substantially lower palmitic acid intake among children compared with a decade ago, consistent with the reduced use of palm oil in processed foods. Children adhering to a GFD, previously considered nutritionally at risk, exhibited a more balanced dietary profile and more favorable metabolic indicators than those reported in earlier cohorts, particularly in the context of nutritional education and regular follow-up visits.

As attention increasingly focuses on the gut barrier, microbiota, and nutritional strategies in the management of gluten-related disorders, these findings suggest that dietary quality should be considered alongside gluten exclusion when promoting healthy gluten-free eating patterns. Emphasizing unsaturated fats, fiber-rich plant foods, and minimally processed ingredients may improve nutritional quality and support cardiometabolic health.

Continued investment in food labeling transparency, dietary education, and public health monitoring is essential to sustain these positive trends. Further longitudinal studies are needed to clarify the long-term clinical implications of evolving dietary patterns in children following a GFD.

## Data Availability

The raw data supporting the conclusions of this article will be made available by the authors.

## Footnotes

1
https://www.winfood.it/software-dieta-professionale/
